# A Newer Technique of Distal Ulna Reconstruction Using Proximal Fibula and TFCC Reconstruction Using Palmaris Longus Tendon following Wide Resection of Giant Cell Tumour of Distal Ulna

**DOI:** 10.1155/2013/953149

**Published:** 2013-12-24

**Authors:** Elango Mariappan, Pragash Mohanen, Justin Moses

**Affiliations:** Department of Orthopaedics, Sri Manakula Vinayagar Medical College and Hospital, Pondicherry 605 107, India

## Abstract

Giant cell tumour of the bone (GCT) is a rare locally aggressive primary bone tumour with an incidence of 3% to 5% of all primary bone tumours. The most common location for this tumour is the long bone metaepiphysis especially of the distal femur, proximal tibia, distal radius, and the proximal humerus. Involvement of distal ulna is rare accounting for 0.45% to 3.2%. Considering local aggressive nature and high recurrence, wide resection is the treatment recommended. Instability of ulnar stump and ulnar translation of the carpals are known complications following resection of distal ulna. To overcome these problems, we attempted a newer technique of distal ulna reconstruction using proximal fibula and TFCC reconstruction using palmaris longus tendon following wide resection of giant cell tumour of distal ulna in a 44-year-old male. This technique of distal radioulnar joint reconstruction has excellent functional results with no evidence of recurrence after one-year followup.

## 1. Introduction

Giant cell tumour of the bone (GCT) is a rare locally aggressive primary bone tumour with an incidence of 3% to 5% of all primary bone tumours [1]. It generally occurs in adults between the ages of 20 and 40 years with slight female preponderance. The most common location for this tumour is the long bone metaepiphysis especially of the distal femur, proximal tibia, distal radius, and the proximal humerus. Involvement of distal ulna is rare accounting for 0.45% to 3.2% [2]. As most of these tumours are locally aggressive in nature, wide resection of the distal ulna is the recommended treatment for GCTs in such locations [3]. The loss of ulnar support results in wrist instability leading to pain, weakness, and loss of grip strength as the ulnar stump may impinge upon the distal radius [4–6]. To overcome this limitation, various reconstructive procedures have evolved. Some authors have reported successful outcome following extensor carpi ulnaris (ECU) tenodesis of the distal stump [7]. A satisfactory outcome has also been reported after the placement of radioulnar prosthesis [8]. Some authors have combined the extensor carpi ulnaris tenodesis with iliac crest graft to the distal radius [9, 10]. We report a case of giant cell tumour of the distal ulna in a 44-year-old male treated by wide resection and reconstruction of the distal radioulnar joint (DRUJ) with proximal fibula and triangular fibrocartilage complex (TFCC) reconstruction using palmaris longus graft with augmentation by extensor carpi ulnaris tenodesis and stabilisation of the graft with dynamic compression plating.

## 2. Case Report

**Figure 1:**
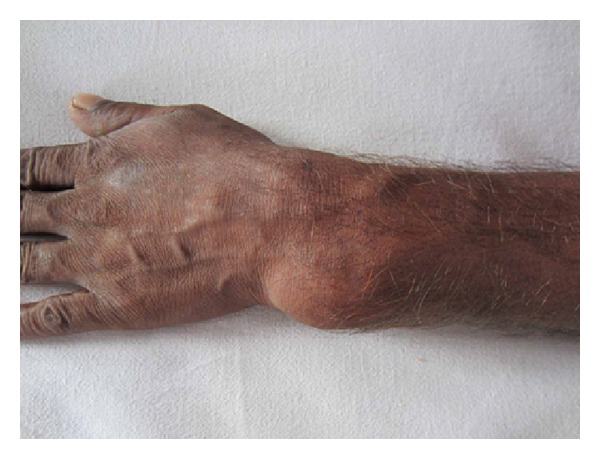
Preoperative clinical photograph.

**Figure 2:**
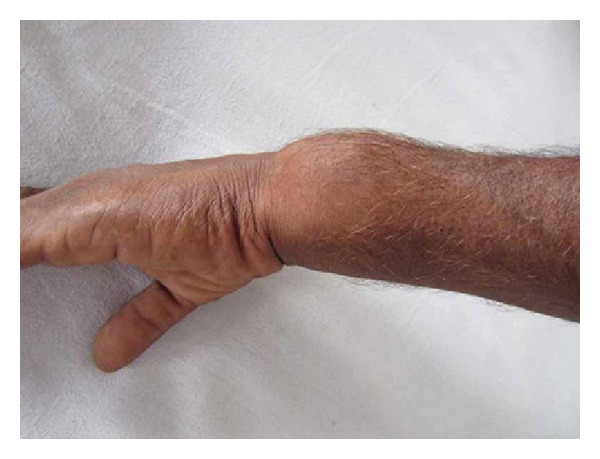
Preoperative clinical photograph.

**Figure 3:**
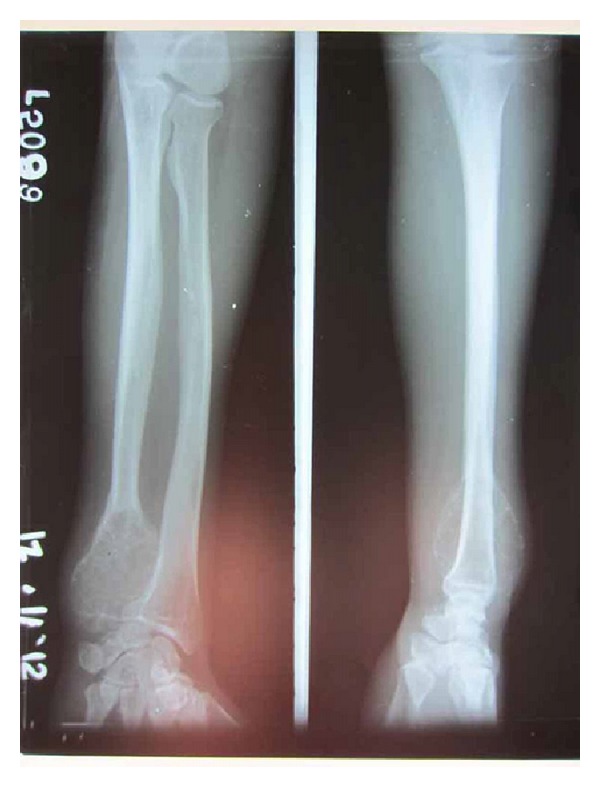
Preoperative X-ray.

**Figure 4:**
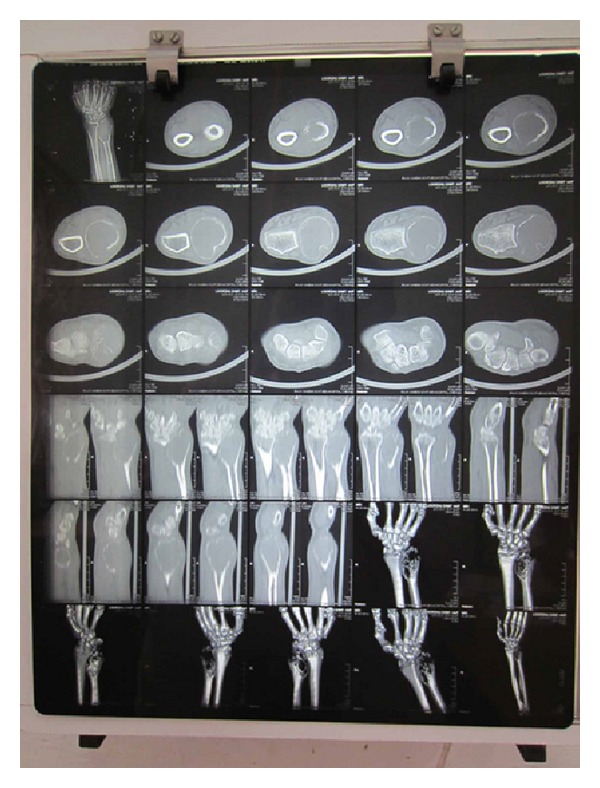
Preoperative computerised tomography.

**Figure 5:**
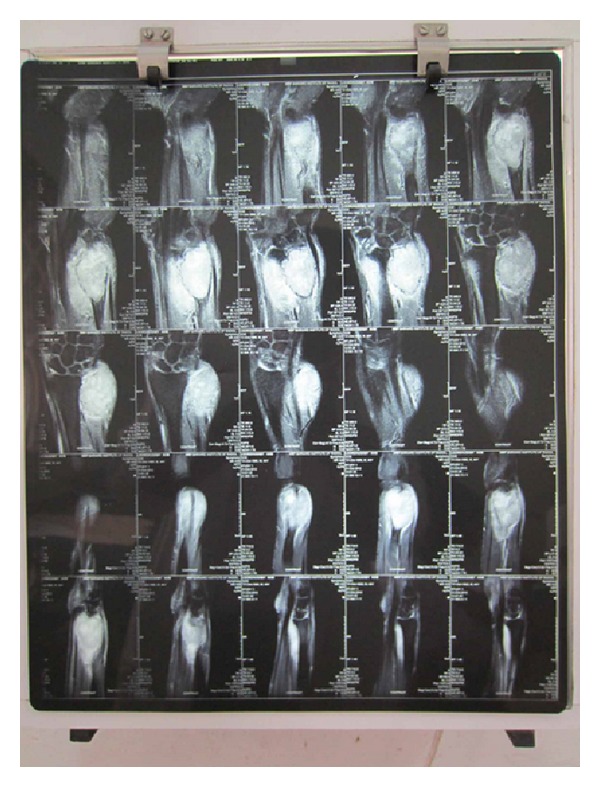
Preoperative MRI.

A 44-year-old male, manual labourer by occupation, presented to our outpatient department with complaints of pain and swelling over the left wrist for the past two years. The swelling was initially small to begin with but gradually grew to the present size. Pain was initially intermittent and was present during strenuous activities, but now there was constant dull aching pain even at rest. There was no history of trauma or constitutional symptoms like fever, loss of weight, or loss of appetite or no associated swellings elsewhere in the body. Examination revealed a firm to hard oval swelling over the distal ulna measuring 5 cm by 4 cm (Figures 1 and 2). Skin over the swelling was normal. Tenderness was present on deep palpation. Terminal restriction of flexion and extension of wrist was noted. Routine serum biochemical studies were within normal limits. Plain radiography of the wrist in anteroposterior and lateral views showed a large expansile multiloculated lesion in the distal ulna with cortical thinning and no periosteal reaction (Figure 3). No evidence of calcification was noted. CT scan of the wrist showed expansile lytic lesion with cortical thinning and few areas of cortical destruction (Figure 4). MRI of wrist revealed 6.5 × 5.6 × 5 cm lesion isointense in T1 weighted and hyperintense in T2 weighted image in the distal ulna. The lesion showed enhancement on contrast MRI. Cortical break was noted (Figure 5). Plain radiograph of the chest was normal. Fine needle aspiration cytology of the lesion showed a double cell population with stromal cells and multinucleated giant cells suggestive of giant cell tumour. Clinicoradiologically a provisional diagnosis of giant cell tumour of distal ulna Enneking stage III was made.

As per the staging system, we planned for wide resection of ulna. Anticipating the loss of long segment of ulna, ulnar reconstruction was planned. Reviewing the literature, extensor carpi ulnaris tenodesis of the stump was found to produce good outcome with limitations in pronation-supination movements. Some authors have tried ulnar buttress arthroplasty using iliac crest graft with limitations in movements. In order to overcome these limitations, we planned for reconstruction using proximal fibula and reconstruction of triangular fibrocartilage complex using palmaris longus tendon.

**Figure 6:**
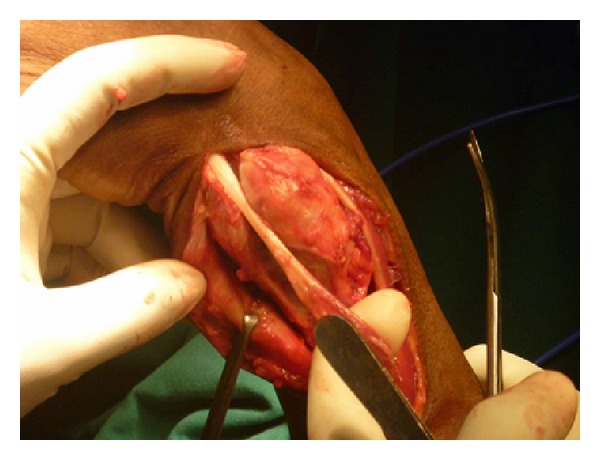
ECU tendon isolated.

**Figure 7:**
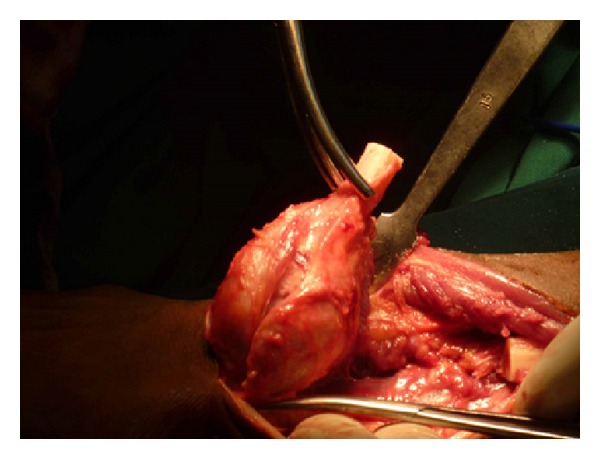
Distal ulna resected.

**Figure 8:**
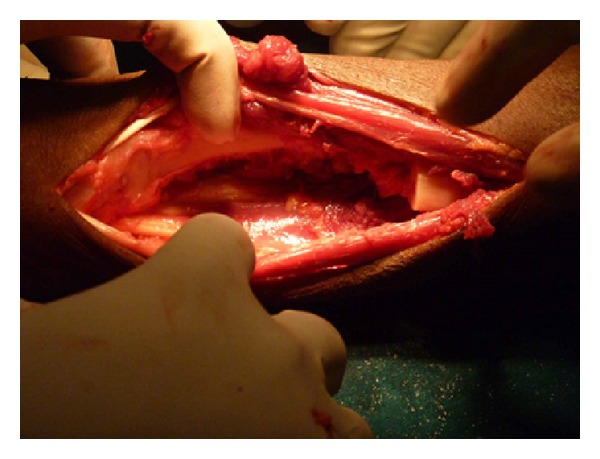
After resection.

**Figure 9:**
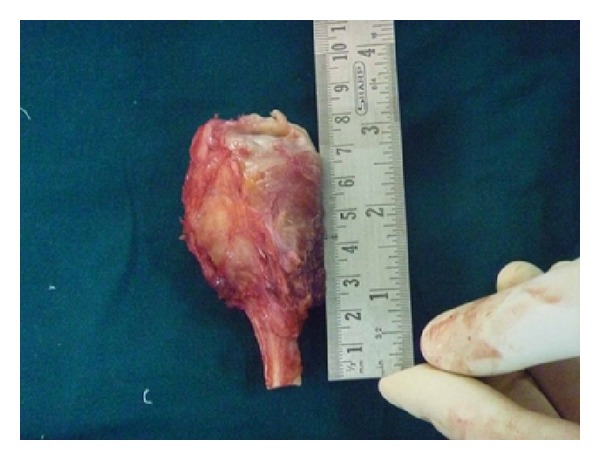
Resected specimen.

**Figure 10:**
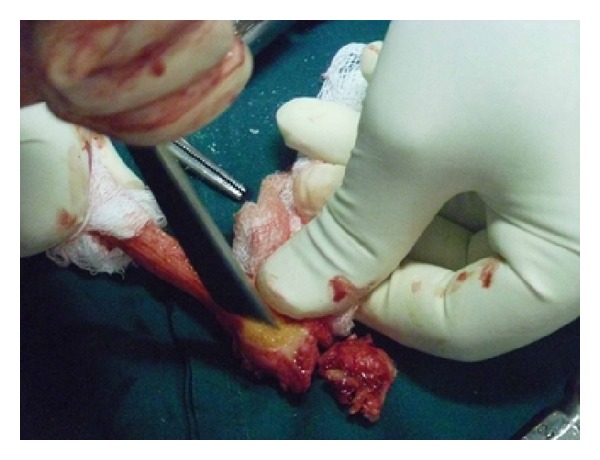
Harvested proximal fibula being trimmed.

**Figure 11:**
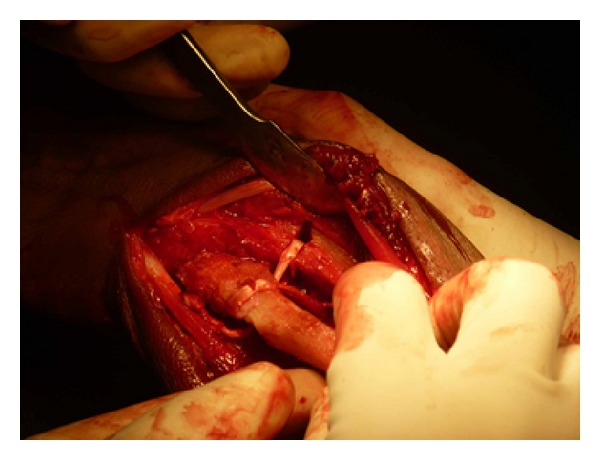
Free palmaris graft for tenodesis of DRUJ.

**Figure 12:**
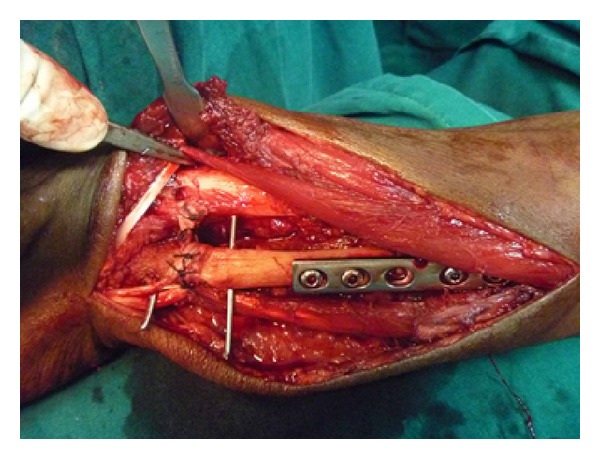
After distal ulna reconstruction.

**Figure 13:**
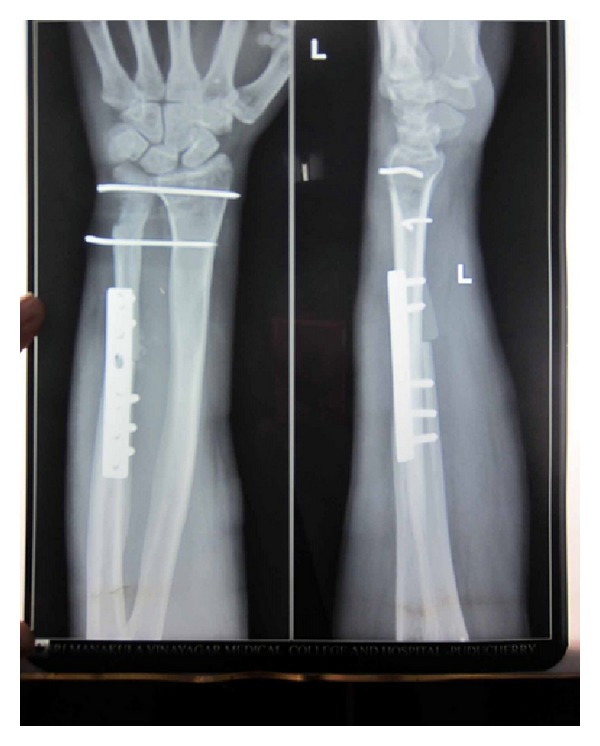
Immediate postoperative X-ray.

Patient was taken up for surgery under combined supraclavicular block and spinal anaesthesia. Through a dorsal approach over the radial border of ulna, wide resection of distal ulna was performed (Figures 6, 7, and 8). The resected ulna measured 8 cm (Figure 9). Around 10 cm of proximal fibula was harvested in routine fashion. The harvested graft was trimmed to fit the distal ulna (Figure 10). Care was taken to position the cartilage surface of fibula facing the radius while the raw surface facing medially as otherwise fusion of the newly constructed DRUJ could occur. The fibular graft was stabilised on the ulnar stump with a 6 holed 3.5 mm narrow dynamic compression plate with 5 screws. To stabilise the distal radioulnar joint, palmaris longus tendon free graft was harvested through two separate stab incisions, one at the level of wrist and the other in the proximal forearm on the volar surface. A drill hole was made across the joint. Palmaris longus tendon was passed through the hole and sutured back on to it (Figure 11). To protect the palmaris longus tenodesis, two K wires were drilled additionally across the DRUJ. To augment the tenodesis, a slip of ECU was sutured to the palmaris longus tenodesis (Figures 12 and 13). Wounds were closed in routine fashion and above elbow POP slab was applied with forearm in supination. Sutures were removed on the 12th postoperative day. Histopathological examination of the resected specimen was consistent with giant cell tumour.

**Figure 14:**
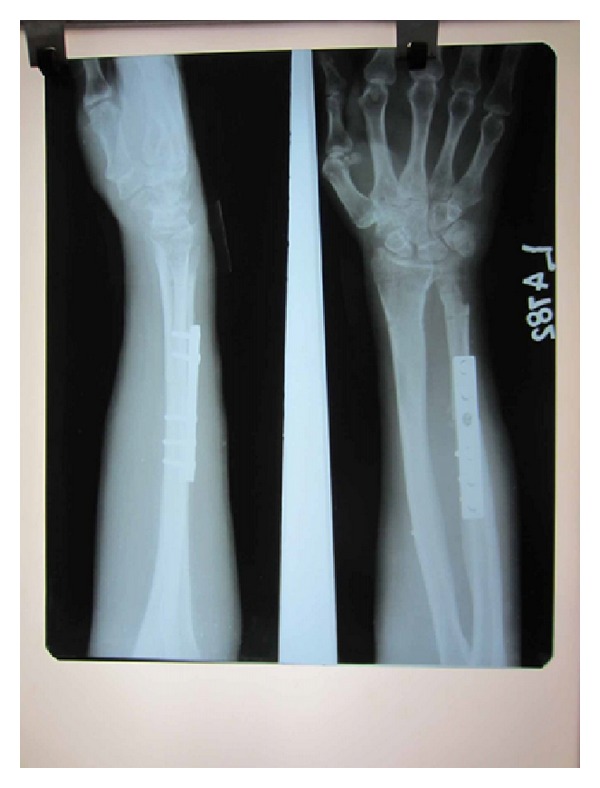
X-ray at 1-year followup.

**Figure 15:**
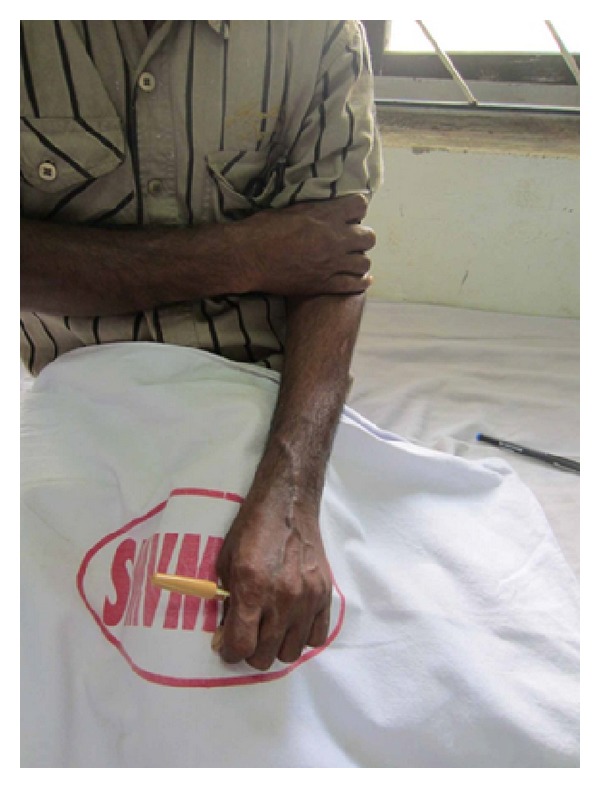
ROM (pronation) at 1-year followup.

**Figure 16:**
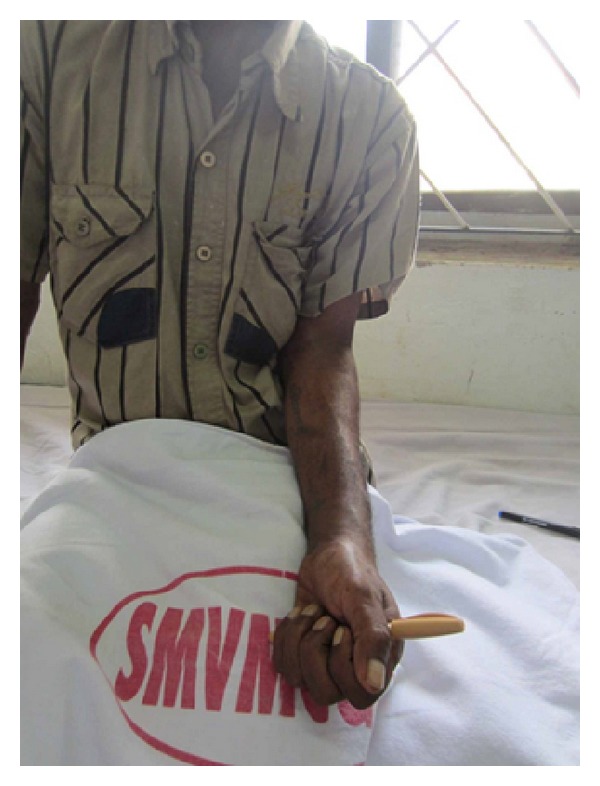
ROM (supination) at 1-year followup.

Strict immobilisation was continued for 6 weeks. At the end of the 6 weeks, the K wires were removed and a full range of movements were initiated. Patient was followed up monthly for the first 6 months. Radiographic and clinical evaluation at the end of 1 year showed good union with no subluxation of the newly created DRUJ and ulna (Figure 14). A near normal range of movements of the wrist including pronation and supination were possible and painless with a good hand grip (Figures 15 and 16). Patient was able to do routine activities since then. Patient had returned to normal work with no evidence of recurrence either clinically or radiologically.

## 3. Discussion

Giant cell tumour of the bone accounts for only 3–5% of all primary bone tumours [1]. The commonest location is the metaepiphysis of distal femur, proximal tibia, distal radius, and proximal humerus. GCT of distal ulna is rarer accounting for only 0.45–3.2% [2]. Most of these lesions are presented either as Enneking stage II or stage III lesions. Because of their aggressive nature with a high potential for recurrence, wide excision is recommended. Traditionally, distal ulna has been considered as a dispensable bone. Darrach effectively resected the distal ulna while dealing with degenerative conditions of DRUJ. Since then, Darrach's procedure and its modification by Dingman [11] have been one of the treatment options for degenerative conditions of DRUJ. However the failure rate for such procedures has been reported to be as high as 10–50% [11]. Further the distal end of the ulna is functionally important as it helps in pronation-supination of forearm and grip strength and in maintaining the relationship between the carpus and distal end of the radius through the ulnar collateral ligament and TFCC [12].

Most of the initial studies on GCT ulna recommended wide resection of the distal ulna. Cooney et al. achieved excellent results in 75% of the cases treated by wide resection alone and concluded that osseous reconstruction is not routinely indicated [13]. But many authors believe that wide resection in tumourous conditions may not be functionally equivalent to the excision in Darrach's procedure which was meant for degenerative conditions. Significant soft tissue loss and bone loss are encountered in tumour resection surgery leading to instability of ulnar stump.

To overcome this problem, focus has been shifted to reconstruction or stabilisation of the ulnar stump. Gainor described a “lasso” tendon graft stabilisation of the ulnar stump and found excellent results in two patients treated by this method [14]. In a series of nine cases, Ferracini et al. performed a soft tissue stabilisation procedure in seven cases using flexor carpi ulnaris, fascia lata, or an autograft and without reconstruction in two cases. All the seven cases with reconstruction had excellent outcome while the two cases without reconstruction had a fair outcome [5]. Kayias et al. utilised extensor carpi ulnaris for tenodesing the distal ulnar stump and reported excellent oncological and functional outcome [7]. Hashizume et al. described the ulnar buttress arthroplasty using autogenous iliac crest bone graft and reported good oncological and functional outcome [15]. Some authors have combined the extensor carpi ulnaris tenodesis with iliac crest graft to the distal radius [9, 10]. More recently, Roidis et al. achieved good functional outcome after distal ulnar implant arthroplasty as a definitive treatment for a recurrent GCT of distal ulna [6].

To our knowledge, reconstruction of the entire resected ulna using proximal fibula combined with DRUJ stabilisation using palmaris longus tenodesis has never been reported in the literature. Most of the studies showed good to excellent functional outcome but invariably with limitation of pronation-supination. In our technique, reconstruction of TFCC by palmaris longus tenodesis and reconstruction of ulna using proximal fibula effectively created a new DRUJ, thereby allowing near normal range of motion including pronation-supination. ECU tenodesis was used only to augment the palmaris longus tenodesis and not to stabilise the proximal ulnar stump as reported in the literature.

## 4. Conclusion

Giant cell tumour of distal ulna is a rare entity with no clear-cut guidelines for treatment. As most of these tumours are locally aggressive in nature, wide resection is the treatment of choice. Most authors would agree that, following resection, some form of stabilisation of ulnar stump is mandatory to provide good functional outcome.

Wide resection of the tumour followed by reconstruction of resected ulna using proximal fibula fixed with a dynamic compression plate combined with ECU tenodesis to prevent ulnar subluxation and TFCC reconstruction by palmaris longus tenodesis is an effective treatment option for GCT of distal ulna.
